# Time-to-Treatment Delays and Their Prognostic Implications in Pharyngeal Cancer—An Exploratory Analysis in Western Romania

**DOI:** 10.3390/clinpract14040103

**Published:** 2024-06-29

**Authors:** Andreea Mihaela Kiș, Roxana Buzatu, Lazar Chisavu, Marioara Poenaru, Claudia Borza, Andrada Iftode, Oana Silvana Sarau, Cristina Adriana Dehelean, Simona Ardelean

**Affiliations:** 1 Faculty of Pharmacy, “Victor Babeș” University of Medicine and Pharmacy, Eftimie Murgu Square No. 2, 300041 Timișoara, Romania; kis.andreea@umft.ro (A.M.K.); andradaiftode@umft.ro (A.I.); cadehelean@umft.ro (C.A.D.); 2 Research Center for Pharmaco-Toxicological Evaluations, Faculty of Pharmacy, “Victor Babeș” University of Medicine and Pharmacy, 300041 Timișoara, Romania; 3 Department of Dental Aesthetics, Faculty of Dental Medicine, “Victor Babeș” University of Medicine and Pharmacy, 300041 Timișoara, Romania; 4 Department of Nephrology, “Victor Babeș” University of Medicine and Pharmacy, 300041 Timișoara, Romania; chisavu.lazar@umft.ro; 5 Department of ENT, “Victor Babeș” University of Medicine and Pharmacy, 300041 Timișoara, Romania; poenaru.marioara@umft.ro; 6 Department of Functional Sciences–Pathophysiology, “Victor Babeș” University of Medicine and Pharmacy, 300041 Timișoara, Romania; borza.claudia@umft.ro; 7 Centre for Translational Research and Systems Medicine, “Victor Babeș” University of Medicine and Pharmacy, 300041 Timișoara, Romania; 8 Department of Hematology, “Victor Babeș” University of Medicine and Pharmacy, 300041 Timișoara, Romania; oana.sarau@umft.ro; 9 Department of Pharmaceutical Technology, Faculty of Pharmacy, “Vasile Goldiș” Western University, 86 Liviu Rebreanu, 310045 Arad, Romania; ardelean.simona@uvvg.ro

**Keywords:** time to treatment, pharyngeal cancer, overall survival, delayed diagnosis

## Abstract

Background: Diagnosis and treatment for pharyngeal cancer are decisive in determining prognosis. Diagnosis delays are frequent, representing a significant cause of avoidable mortality, and an important factor in subpar survival across the continuous HNC care delivery. Methods: The present study represents a retrospective analysis of medical records from Western Romania, which included 180 patients, to evaluate the impact of time-to-treatment delay on patients diagnosed with pharyngeal cancer. The data analyses were performed using the Kaplan–Meier method R (version 3.6.3) packages, including tidyverse, final-fit, mcgv, survival, stringdist, janitor, and Hmisc. Results: The mean days from diagnosis until the end of treatment were higher for the nasopharynx group. Cox regression analysis regarding diagnosis to treatment duration categories showed an increased risk mortality by 3.11 times (95%CI: 1.51–6.41, *p =* 0.0021) with a Harrell’s C-index of 0.638 (95%CI: 0.552–0.723). The hypopharynx and oropharynx locations increased risk mortality by 4.59 (95%CI: 1.55–13.55) and 5.49 times (95%CI: 1.79–16.81) compared to the nasopharynx location. Conclusions: The findings of this study led to the conclusion that it seems there is a trend of mortality risk for oropharynx and hypopharynx cancers due to delays in the time to treatment over 70 days, standing as a basis for further research as there is an imperative need for prospective multicenter studies.

## 1. Introduction

Head and neck cancer (HNC) incidence is increasing worldwide, and the risk factors are usually tobacco smoking, alcohol consumption, Human Papillomavirus (HPV), especially types 16 and 18, and infection with Epstein–Barr Virus (EBV) [1,2]. These tumors are distinguished by their quick growth in crucial anatomic regions. Their treatment significantly influences vital processes like breathing, speech, and swallowing [3]. 

The pharynx is a muscular cavity that begins behind the nose and ends in the larynx and esophagus [4]. It has three different anatomical parts: the nasopharynx, which is situated in the upper part of the pharynx posterior of the choanae; the oropharynx, which begins from the posterior wall of the palate and ends at an imaginary line at the top of the external margin of the epiglottis; and the hypopharynx, which is situated posteriorly from the larynx and is comprised of the two pyriform sinuses (left and right), the posterior pharyngeal wall, and the posterior part of the cricoid [4]. The most common type of pharyngeal cancer is squamous cell carcinoma [5]. The symptomatology of pharyngeal cancer is not particular and it is usually confused with pharyngitis symptoms or even with a viral etiology of tonsillitis [6,7]. The most common symptoms refer to sore throat, dysphagia, and odynophagia, which may be another cause for diagnosis and treatment delay; reflex earache is found in nasopharyngeal cancer [6,8]. Unfortunately, the first noticeable symptomatology that appears is lymphadenopathy [9]. 

One of the primary therapeutic choices for early tumors is surgical excision followed by radiation, while for advanced tumors, it is radiation therapy (RT), which is used in doses ranging from 54 to 70 Gy according to a typical fractionation schedule of 2 Gy/fraction, one fraction/day, and five fractions/week. Furthermore, the usual nonsurgical strategy in high-risk situations is to combine RT with concurrent cisplatin (100 mg/m^2^ every three weeks) [10]. As radiation therapy advances, more complex treatment methods are available. Most centers currently employ conformal radiation treatment (CFRT), and intensity-modulated radiation therapy (IMRT) is also becoming more widespread [11]. However, despite all these newly developed technologies, the delayed treatment influences the dosage due to cancer cells spreading in the whole organism, causing higher toxicity due to the side effects of the employed radiotherapy [12].

Diagnostic delays are also frequent, representing a significant cause of avoidable mortality, and an important factor in subpar survival across continuous HNC care delivery [13,14]. These delays are usually divided into two parts: (i) patient delay, indicating the time frame from when the first symptoms appear and the patient searches for the first specialist appointment, and (ii) system delay, covering the time from the first specialist appointment to the histopathological diagnosis and treatment initiation (DTI) [15]. Due to the advanced stages of the diagnosis of these tumor types, the initiation of the treatment is important for a better outcome [13]. While time of diagnosis to treatment intervals (TDTIs) can vary in their effects on overall survival, extensive studies have shown that longer TDTIs have detrimental impacts on treatment progression [14]. Regarding HNC time treatment: the diagnostic to treatment interval (DTI) and treatment package time (TPT), which refers to the time of the first specialist’s consultation to the end of postoperative radiotherapy, have been evaluated in the United States but not widely researched in Europe [3,14,16]. In the Netherlands, the indicator norm for treatment initiation was set at 30 days [17], similar to other international studies [18,19,20]. 

To date, in Romania, as well as in other Eastern European countries, the impact of treatment time delay on the prognosis of pharyngeal cancer remains poorly investigated. To the best of our knowledge, this is the first study conducted in the western region of Romania that aims to explore the potential influence regarding the increase in DTTIs on pharyngeal cancer-diagnosed patients’ survival undergoing chemo- or radiotherapy.

## 2. Materials and Methods

### 2.1. Study Design

This study is a retrospective analysis of medical records from the Timisoara Municipal Emergency Clinical Hospital. We reviewed the records of patients over 18 years of age who underwent primary surgical interventions (biopsies) for pharyngeal malignant tumors between 1 January 2014 and 31 December 2018.

The inclusion criteria were as follows: patients with previously untreated pharyngeal carcinoma that was located on the nasopharynx, oropharynx, and hypopharynx; patients who completed curative-intent surgery and full-course adjuvant radiation; and patients who received definitive surgical management and had a 48-month follow-up (or until death).

Exclusion criteria: patients who received treatment for their tumor elsewhere (different center); patients with a previous history of HNC; patients with the absence of evidence of treatment or follow-up; patients with prior chemo- or radiotherapy in the head and neck area; and patients who had uncertain, zero, or negative time intervals from diagnosis to therapy, surgical recovery, and radiation treatment.

#### Definitions

- Diagnosis to treatment interval (DTI): time from histopathological diagnosis to the start of treatment.
- Treatment package time (TPT): time from the first day with the specialist to the end of curative treatment.

The final group selected for the study comprised 180 patients with previously untreated HNC.

### 2.2. Diagnosis Process

All patients underwent a comprehensive diagnostic evaluation with a full medical examination and detailed medical history followed by a physical examination. Imaging and pathological procedures included chest radiography or chest CT (performed in cases of suspicious low cervical lymph nodes or bilateral cervical lymph nodes); biopsy of lymph nodes suspected of metastases; and head and neck imaging using computed tomography (CT) and/or magnetic resonance imaging (MRI).

Tumor staging was categorized using the TNM Classification of Malignant Tumors 8th edition.

### 2.3. Data Collection

The study focused on patients diagnosed with pharyngeal cancer and treated at Timisoara Municipal Emergency Clinical Hospital. Specific data recorded included:

- Initial diagnostic of tumor pathology.
- Age at diagnosis.
- Gender.
- Residency environment (rural or urban).
- Tumor localization (nasopharynx, oropharynx, hypopharynx).
- Type of hospitalization (emergency or routine).
- Surgical intervention details.
- Biopsy type and histological diagnosis.
- Details of the patient’s first admission to the ENT ward and oncological treatment.
- Details on adjuvant treatments and anti-neoplastic therapies, along with the prognosis considering the timing of initiation for chemotherapy/radiotherapy and the status of patients (alive or deceased).

### 2.4. Time Intervals Defined

- First presentation to diagnosis interval: time from the first consultation within the Timisoara Municipal Emergency Clinical Hospital to the final histopathological diagnosis.
- Diagnosis to treatment interval (DTI): time from the final diagnosis to the start of treatment (either the surgery date or the first fraction of chemoradiotherapy).

### 2.5. Outcome Measurements

The primary endpoints of this study encompassed: oncological outcomes with an assessment of the disease’s progression, status, and prognosis; the impact of delays’ evaluation and how this influences oncological outcomes; and time interval measurements of the duration between the initial consultation to diagnosis and subsequent treatment initiation.

For calculating follow-up, recurrence rates, and disease-specific survival, we relied on the findings from the most recent outpatient examinations conducted by the surgeon (head and neck) or oncologist. Overall survival was determined based on the date of the last contact with the patient or the date of their death.

### 2.6. Ethical Statement

The current study was evaluated and approved by the Scientific Research Ethics Committee of Victor Babes University of Medicine and Pharmacy, Timisoara (Approval No. 53/28 September 2018).

### 2.7. Statistical Analysis

The survival functions were estimated with the Kaplan–Meier method, and differences were assessed using a log-rank test. Data were collected and analyzed using R (version 3.6.3); the packages employed include tidyverse, final-fit, mcgv, survival, stringdist, janitor, and Hmisc. The continuous variables were presented both with Gaussian distributions: means ± standard deviation (SD), as well as without Gaussian distribution: medians and inter-quartile range, and the categorical variables were presented as percentages.

The statistical tests used were: for Gaussian populations, the Student’s *t*-test or analysis of variance for means; for non-Gaussian populations, the Mann–Whitney U test or the Kruskal–Wallis test for medians; and for proportions, an χ^2^ test. Continuous variable distributions were assessed for normality using the Shapiro–Wilk test and for equality of variance using Levene’s test. Association strength between non-Gaussian continuous variables was gauged using Spearman’s correlation coefficient. A *p*-value of <0.05 was set as the threshold for statistical significance.

## 3. Results

### 3.1. Patient Retrospective Observational Study 

Out of 515 patients evaluated between 2014 and 2018, 350 were diagnosed with a malignant pharyngeal tumor. Among these, only 180 met the study criteria. The follow-up time had a median of 6.21 years, IQR: 4.62–7.66 years. The average age of the patients receiving post-biopsy treatments was 59.0 years, with a standard deviation of 9.2 years, and their ages ranged from 31.0 to 81.0 years. Most patients were males, accounting for 94.4%, while females constituted 5.6%. 

In terms of histopathological diagnosis and treatments, squamous cell carcinoma (SCC) was identified in 67.2% of patients (121 in total). Of these, 67.8% were treated with radiotherapy (RxT), 24 patients with chemoradiotherapy (CRT), and 15 patients with chemotherapy (ChT). Keratinizing squamous cell carcinoma (KSCC) was diagnosed in 16.1% of patients (29 in total), with 17 undergoing RxT, 7 receiving CRT, and 5 being treated with ChT. The N staging revealed that 53.9% of patients were at the N0 stage, with 71 out of 121 undergoing RxT. For the T2 and T3 stages, 44.6% (54 patients) and 20.7% (25 patients) were treated with RxT, respectively. The detailed clinical characteristics of the population are displayed in Table 1.

Table 1 provides a detailed portrayal of the patients’ retrospective demographics, histopathologic diagnosis, intervals from symptom recognition to seeking medical care, the extent of lymph node involvement, tumor staging, and surgical interventions. The table facilitates a thorough understanding of the patients and the diverse treatment pathways. However, the *p*-values suggest that the differences regarding the treatment modality of groups may not be statistically significant. This comprehensive table is a reference for the analysis of treatment modalities and outcomes in the context of demographic and clinical parameters.

### 3.2. Treatment Intervals

The median duration time from the first appointments of the patients with a specialist until histopathological diagnosis was 9 days. We set up a reference level of 14 days for this interval, and no significant detriment was observed when comparing intervals exceeding this reference.

The median DTI was 33 days. Furthermore, the median TPT was 100 days, ranging from 28 to 2217 days, with a standard deviation of 271 days. The mean days from diagnosis until the end of treatment were notably higher for the nasopharynx group, but this difference was not statistically significant. In the multivariable analysis of this retrospective study, DTI did not show a significant impact (Table 2).

**Figure 1:**
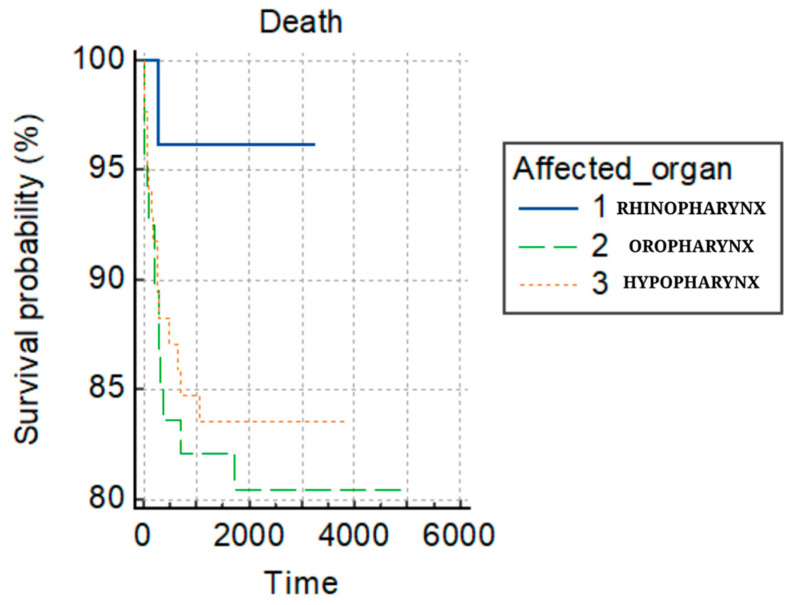
Survival probability of death.

Regarding treatment modalities, chemotherapy was administered to 32.8% of patients, while 87.8% underwent radiotherapy. The overall mortality rate stood at 16.66%, with only one patient from the nasopharynx group succumbing to the disease. The rate of disease recurrence was 26.1%. Most patients (54.4%) presented at the N0 stage when assessing adenopathy classifications. The nasopharynx group showed the highest incidence (38.5%) of N3 adenopathy. In this retrospective study, 42.2% of patients began treatment within 30 days post-diagnosis. However, the oropharynx and hypopharynx groups had the most treatment delays, and, according to Figure 1, they also presented an increased mortality risk. The delays in our group regarding the day’s diagnosis to the end of treatment are high. Moreover, for the oropharynx and hypopharynx groups versus the nasopharynx group, diagnosis to treatment was prolonged (Table 3).

As one can see from Figure 1, the hypopharynx and oropharynx locations increased the risk of death by 4.59 (95%CI: 1.55–13.55) and 5.49 times (95%CI: 1.79–16.81) compared to the nasopharynx location.

### 3.3. Oncological Treatment

The retrospective group of 180 patients who underwent various treatment modalities is categorized as follows:

(a) ChT (chemotherapeutic treatment): This group received combined therapies which included:
  ○ Single-agent regimens: carboplatin (40 mg/m^2^ IV weekly for 6–7 wks), docetaxel (75 mg/m^2^ IV every 3wk), and methotrexate (40 mg/m^2^ IV weekly (3 wks equals one cycle).
  ○ Dual-agent combinations: paclitaxel (200 mg/m^2^ IV every 3 wks) with carboplatin (40 mg/m^2^ IV weekly for 6–7 wks); cetuximab (400 mg/m^2^ IV loading dose 1 wk before the start of radiation therapy, then 250 mg/m^2^ weekly) with carboplatin (40 mg/m^2^ IV weekly for 6–7 wks); docetaxel (75 mg/m^2^ IV every 3 wks) with either cisplatin (100 mg/m^2^ IV on days 1, 22, and 43 or 40 mg/m^2^ IV weekly for 6–7 wks) or carboplatin (40 mg/m^2^ IV weekly for 6–7 wks); as well as 5-Fluorouracil (5-FU 800 mg/m^2^ by continuous IV infusion on days 1–5 given on the days of radiation) with cisplatin (100 mg/m^2^ IV on days 1, 22, and 43 or 40 mg/m^2^ IV weekly for 6–7 wks).
  ○ Triple-agent combinations: paclitaxel (200 mg/m^2^ IV every 3 wks) combined with carboplatin (40 mg/m^2^ IV weekly for 6–7 wks) and cetuximab (400 mg/m^2^ IV loading dose 1 wk before the start of radiation therapy, then 250 mg/m^2^ weekly) or epirubicin (100 mg/m² IV); and docetaxel (75 mg/m^2^ IV every 3 wks) combined with carboplatin (40 mg/m^2^ IV weekly for 6–7 wks) or cisplatin (100 mg/m^2^ IV on days 1, 22, and 43 or 40 mg/m^2^ IV weekly for 6–7 wks) and cetuximab (400 mg/m^2^ IV loading dose 1 wk before the start of radiation therapy, then 250 mg/m^2^ weekly).
(b) RxT (radiotherapy): Patients were treated with cobalt therapy, often in conjunction with cisplatin, intensity-modulated radiation therapy (IMRT), and three-dimensional conformal radiation therapy (3D CRT). In high-risk patients, it was used: 60–66 Gy (2.0 Gy/fraction), daily Monday–Friday for 6–6.5 weeks; and for those at intermediate risk: 44–50 Gy (2.0 Gy/fraction) to 54–63 Gy (1.6–1.8 Gy/fraction).
(c) CRT (chemoradiotherapy): this group received a combined treatment based on chemotherapy and radiotherapy.

**Figure 2:**
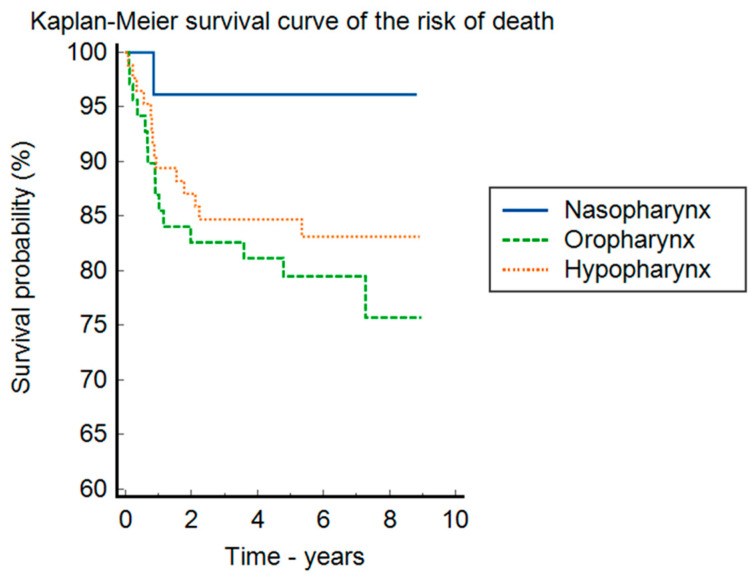
The Kaplan–Meier survival curve in the risk of death, stratified by the location of the neoplasia.

**Figure 3:**
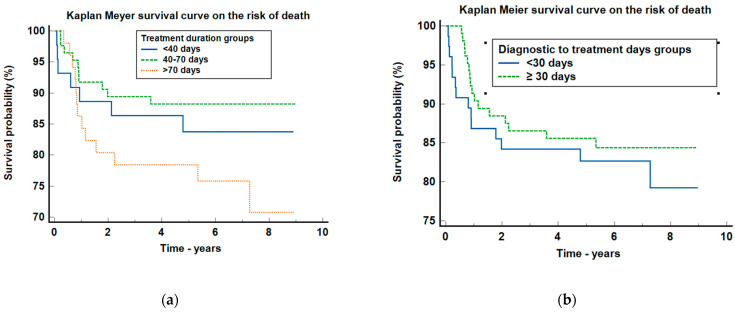
Graphical representation of the Kaplan–Meier survival curve on the risk of death for (**a**) treatment duration groups and (**b**) diagnostic to treatment days groups.

The detailed clinical characteristics of this patient group can be found in Table 4 and the Kaplan–Meier analyses in Figure 1, Figure 2 and Figure 3.

We further evaluated patients regarding recurrence. The only statistical difference between groups was the therapy used to treat the initial neoplasia episode. The recurrence patients were often treated with chemo- or chemoradiotherapy rather than radiotherapy.

In the Kaplan–Meier survival curve (Figure 3), regarding the risk of death stratified by the location of the neoplasia, the p-value of the log-rank test is 0.126. There seems to be a trend of increased mortality in the oropharynx (HR = 6.21, 95%CI = 2.1–18.3) compared to the nasopharynx, and in the hypopharynx (HR = 4.61 95%CI = 1.61–13.14) compared to the nasopharynx.

Regarding the survival curve of the risk of death for treatment duration groups (Figure 3a), the *p*-value was 0.129, meaning that no statistical differences were observed among the groups, but there is a trend of increased mortality between the groups with more than 70 days of treatment duration compared with the ones with 40–70 days, HR = 2.26 (95%CI: 0.96–5.33).

As regards the Kaplan–Meier survival probability for the diagnostic to treatment days groups (Figure 3b), the *p*-value was 0.537, meaning that no statistical differences concerning the risk of death were observed.

**Figure 4:**
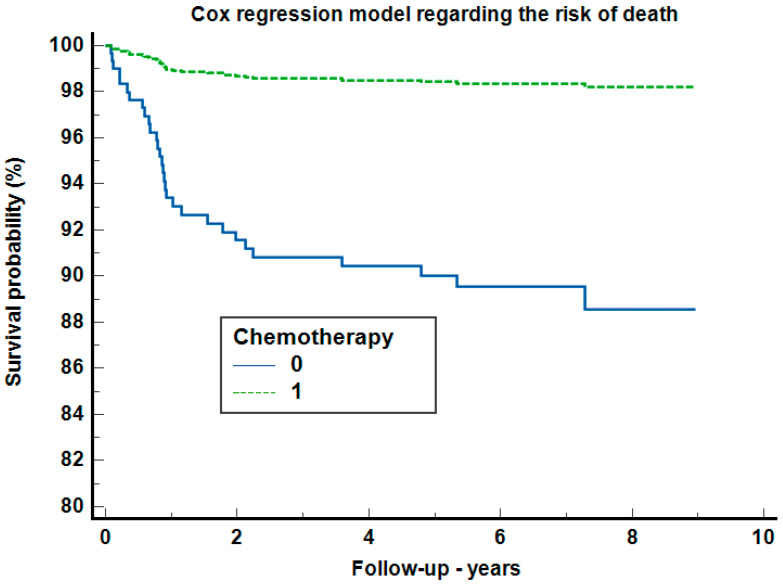
The Cox regression analysis regarding risk mortality.

In the Cox regression analysis regarding risk mortality (Figure 4), after adjustment for adenopathy, radiotherapy, sex, age, localization, and diagnostic to treatment duration categories, only chemotherapy increased the risk of death, by 3.11 times (95%CI: 1.51–6.41, *p =* 0.0021) with a Harrell’s C-index of 0.638 (95%CI: 0.552–0.723).

## 4. Discussion

The present study was conducted to investigate the effects of a delay in diagnosis and treatment on the prognosis of patients diagnosed with pharyngeal cancer in the western region of Romania. The major findings indicated that a time-delayed treatment prolongs the treatment package time and therefore has an unfavorable impact on tumors’ fast growing. The nasopharynx group had the most delayed TPT but with a better outcome than the other two sites. We found a more than 50% delayed DTI, especially for the oropharynx, which increased mortality (HR = 6.21, 95%CI = 2.1–18.3).

The DAHANCA randomized trial research for HNSCC attested that prompt radiotherapy is important for treatment outcome and for overall time treatment (OTT). Nevertheless, the accelerated fractionation applied to HNSCC, regardless of the HPV/p16 status, reveals a better outcome than conventionally fractioned radiotherapy even in cases of rapidly growing tumors [21]. Withers initially demonstrated in the 1980s that extended OTT results in the loss of local control, and that an additional 0.6 Gy/day is needed to offset the faster repopulation of tumor tissue, which indicates that an increasing time delay also affects the treatment dosage, which influences the side effects of radiation treatment [12]. The side effects of increased concentrations and longer treatments are associated with mucositis, dermatitis, dysgeusia, dysphagia, and odynophagia, which can lead to poor oral intake and weight loss. In rare cases, this can even cause discontinuation or stopping therapy and could influence the outcomes [22]. A research study conducted in the United States, which has been focused on optimizing the diagnosis research, found that even after adjusting for comorbidities, age, and stage, it is likely that disease progression (the gap time from diagnosis to treatment >30 days) harms the overall survival due to the need for further treatment or readapting of the treatment plan in some cases [18]. Murphy et al. [3] and van Harten et al. [23] assessed the continued impact of diagnosis to treatment on survival and found that prolonged intervals are correlated with reduced overall survival [3,18,23]. Specifically, Murphy et al. reported diminished patient survival rates when treatment extended beyond 61 days from diagnosis. These authors’ findings are well correlated with the results of our study, which showed that the mortality increased between the group with delayed treatment (>70 days) as compared with the groups with 40–70 treatment days HR = 2.26 (95% CI: 0.96–5.33) [3,18].

The landmark trials for the best radiation-alone protocol are RTOG 90-03, EORTC 22791, and DAHANCA; these trials are used to guide the appropriateness of the RT component of various chemoradiation arms. Still, the improvements in locoregional control achieved by these newer radiation protocols were only 7 to 10% [3,21,22,23]. Moreover, no agreement exists on the best radiation dose fractionation scheme [24]. Our retrospective study showed at the survival analysis on the risk of death, regarding the type of therapy regimen applied that the group treated only with chemotherapy presented a higher mortality rate compared to the group treated only with radiotherapy. This statement coincides with other studies that have demonstrated that in pharyngeal cancer in its early stages, the first choice of treatment represents local surgery followed by radiotherapy, but this type of treatment is appropriate for advanced stages of the disease. In addition, local surgery can be applied concomitantly with systemic chemotherapy, taking into consideration the patient’s status and comorbidities. In cases of advanced disease or with metastases, surgical treatment can be applied where necessary. However, depending on the site of the primary tumor, the surgery can be limited and radiotherapy represents the first choice (in the nasopharynx, this is due to the radiosensitivity and the anatomical progression of the disease) [3,21,22,23,25]. In this study, the oropharynx and hypopharynx had an increased risk of mortality compared to the nasopharynx location. Pharyngeal cancer treatment aims to eradicate tumors while minimizing adverse effects and preserving the quality of life. The treatment strategy is complex, and in this regard, the care protocol is established by tumor characteristics and patient profile. The treatment strategy is planned according to the stage of the disease and should be discussed within an interdisciplinary board consisting of the ENT surgeon, radiation oncologist, and medical oncologist. Patients who have a high risk of developing the disease again have a smoking and alcohol history, and regardless of the aggressive treatment of platinum-based chemotherapy, local recurrences are developed in 30–40% of cases, as well as metastasis [26,27,28,29,30,31,32]. Definitive radiation is preferred for oropharynx tumors due to its superior functional outcomes [33]. Nowadays, HPV+ OPSCC is considered by the American Joint Committee on Cancer (AJCC) as a separate staging system and treatment as compared with HPV–OPSCC [34]. Taking into consideration that p16 OPSCC has a better survival rate, due to the different biology as well as patient characteristics (younger age, less smoking and alcohol history, fewer comorbidities with a better treatment tolerance), patients will have a better response to chemoradiotherapy [35]. To date, there are no early identification methods for OPSCC screening emerging. As a result, only nationwide HPV vaccination programs can ensure worldwide prevention, and these are already being implemented in Eastern Europe. Regardless of the positive prognosis associated with OPSCC+, 10–25% of patients will develop a recurrence, most of them in the first 2–5 years after diagnosis. Therefore, monitoring the patient after the end of treatment is essential [35]. HPV DNA detection seems like a possibility for the detection of treatment response and measuring the risk of disease progression.

The present study has some limitations such as the p16 analysis not being performed on any of the samples, which precludes a precise quantification of the number of tumors driven by HPV. The lack of p16 analysis represents a significant shortfall since HPV and p16 are recognized for their important prognostics in oropharyngeal squamous cell carcinoma (OPSCC) [34,35,36].

Looking forward to the potential clinical implications of our findings, a superior organization of care in a regional area with a more advanced collaboration between centers and practitioners must be taken into consideration. Moreover, implementing IT solution programs in which the patient’s record files are available in order to reduce repeated diagnosis tests also influences time delay. Regarding tumor sites, it is essential that a better screening program is achieved regarding the appearance of the first signs of symptomatology, especially in oropharyngeal and hypopharyngeal sites. Nevertheless, the patients with expected therapy should benefit from different adjustments that could be implemented on the first day of multidisciplinary consultation including additional scans which may help the radio-oncologist to minimize delays, comorbidity evaluations, malnutrition screening, and even dentistry appointments.

## 5. Conclusions

The present study highlights the evidence regarding the consequences of treatment delay on the prognosis of pharyngeal cancer-diagnosed patients undergoing therapy, offering a comprehensive overview of the cases registered in the western region of Romania between 2014 and 2018. The novel findings revealed a significant correlation between time delay and pharyngeal cancer evolution (especially, oropharynx and hypopharynx cancers). As regards the treatment strategy, the results obtained from the survival analysis on the risk of death stratified by the therapy regimen reveal that applying chemotherapy as treatment led to a higher mortality rate as compared with radiotherapy. In addition, concerning the tumor sites, it seems that oropharynx and hypopharynx cancers may be associated with an increased mortality as compared to nasopharynx cancer. In order to secure a more accurate evaluation, larger studies concerning time delays and outcomes are necessary in prospective multicenter studies. Recurrences are expected to develop in 30–40% of treated patients if they do not follow a healthy lifestyle with improved personal care modalities, related to physical, mental, emotional, and social functioning, which contributes to preserving or increasing the life quality and overall satisfaction. Unfortunately, even the treatment strategies for patients with head and neck cancer play a crucial role in the alteration in quality of life. Therefore, the treatments and protocol interventions should be focused both on survival and on ensuring quality of life. The particularities that should be considered refer to the management of pain, emotional and psychosocial instability, as well as rehabilitation support to overcome the debilitating physical barriers regarding the change in physical appearance, and the loss or modification of certain organs/functions.

In summary, treatment delay is associated with a bad prognosis in patients with head and neck cancer. It is suggested that a direct collaboration with or patient transition to a specialized hospital with improved healthcare capacity available to provide the most innovative treatment approaches represents a good strategy. However, further research should be conducted to verify if this strategy increases survival rate.

## Figures and Tables

**Table 1:** Entire patient retrospective study description (from demographics to treatment).

	ChT N *=* 23	RxT N *=* 121	CRT N = 36	Total N *=* 180	*p*-Value
Age					0.204
Mean (SD)	57.6 (9.5)	59.9 (9.2)	57.1 (8.6)	59.0 (9.2)	
Range	42.0–78.0	31.0–76.0	40.0–81.0	31.0–81.0	
Gender					0.615
M	21.0 (91.3%)	114.0 (94.2%)	35.0 (97.2%)	170.0 (94.4%)	
F	2.0 (8.7%)	7.0 (5.8%)	1.0 (2.8%)	10.0 (5.6%)	
Histopathologic Diagnosis					0.848
SCC	15.0 (65.2%)	82.0 (67.8%)	24.0 (66.7%)	121.0 (67.2%)	
KSCC	5.0 (21.7%)	17.0 (14.0%)	7.0 (19.4%)	29.0 (16.1%)	
SCC IN SITU	1.0 (4.3%)	4.0 (3.3%)	2.0 (5.6%)	7.0 (4.1%)	
UCNT	2.0 (8.7%)	12.0 (9.9%)	3.0 (8.4%)	17.0 (8.4%)	
OTHER CANCERS	0.0 (0.0%)	6.0 (5.2%)	0.0 (0.0%)	7.0 (4.1%)	
Patient delay					0.375
Unspecified	15.0 (65.2%)	56.0 (46.3%)	14.0 (38.9%)	85.0 (47.2%)	
˂3 months	4.0 (17.4%)	39.0 (32.2%)	13.0 (36.1%)	56.0 (31.1%)	
˃3 months	4.0 (17.4%)	26.0 (21.5%)	9.0 (25.0%)	39.0 (21.7%)	
N category					0.329
N0	12.0 (52.2%)	70.0 (57.9%)	15.0 (41.7%)	97.0 (53.9%)	
N1	1.0 (4.3%)	19.0 (15.7%)	7.0 (19.4%)	27.0 (15.0%)	
N2	3.0 (13.0%)	8.0 (6.6%)	5.0 (13.9%)	16.0 (8.9%)	
N3	7.0 (30.4%)	24.0 (19.8%)	9.0 (25.0%)	40.0 (22.2%)	
T Stage					0.071
T1	8.0 (34.7%)	39.0 (32.2%)	13.0 (36.1%)	60.0 (33.4%)	
T2	9.0 (39.1%)	54.0 (44.6%)	10.0 (27.8%)	73.0 (40.6%)	
T3	5.0 (21.7%)	25.0 (20.7%)	9.0 (25.0%)	39.0 (21.7%)	
T4	0.0 (0.0%)	3.0 (2.5%)	2.0 (5.6%)	5.0 (2.8%)	
This	1.0 (4.3%)	0.0 (0.0%)	2.0 (5.6%)	3.0 (1.7%)	
Surgery					0.137
Minor surgery (biopsy)	20.0 (80.9%)	108.0 (89.3%)	32.0 (88.9%)	160.0 (89.0%)	
Major surgery (tracheotomy, cervicotomy, laryngectomy)	5.0 (21.7%)	11.0 (9.1%)	4.0 (11.2%)	15.0 (11.1%)	

Squamous cell carcinoma (SCC), keratinizing squamous cell carcinoma (KSCC), squamous cell carcinoma in situ (SCC IN SITU), and undifferentiated carcinoma of nasopharyngeal type (UCNT). The counts and percentages of patients with each histopathologic diagnosis are provided for each treatment modality group—chemotherapy (ChT), radiotherapy (RxT), chemoradiotherapy (CRT).

**Table 2:** Patients’ interval evaluation.

	Mean (SD)	Median	95% CI for Median	
The first presentation to diagnosis	15.47 (40.08)	9	8–9	180 patients
Diagnosis to treatment (DTI)	68.21 (134.35)	33	29.05–37	175 patients
Radiation interval	112.78 (184.06)	50	48–51.6	180 patients
Treatment package time (TPT)	114.06 (186)	50	48–52	176 patients
The first presentation to diagnosis	15.47 (40.08)	9	8–9	180 patients

Intervals of patients’ medical journey, with statistical measures including the mean, standard deviation, median, and 95% confidence interval for the median provided.

**Table 3:** Nasopharynx, oropharynx, and hypopharynx subgroups’ evaluation.

Parameter	Nasopharynx N = 26 (14.4%)	Oropharynx N = 69 (38.3%)	Hypopharynx N = 85 (47.2%)	Total N = 180	*p*-Value
Days diagnostic–end of treatment M + SD	211 (423.46)	162.36 (177.19)	178.01 (272.15)		0.232
Chemotherapy	7 (26.9%)	21 (30.4%)	31 (36.5%)	59 (32.8%)	0.576
Radiotherapy	23 (88.5%)	62 (89.9%)	73 (85.9%)	158 (87.8%)	
Treatment duration group (Rxt; CRT, ChT)					
<40 days	8 (30.8%)	11 (15.9%)	25 (29.4%)	44 (24.4%)	
40–70 days	10 (38.4%)	41 (59.4%)	34 (40%)	85 (47.2%)	0.1162
>70 days	8 (30.8%)	17 (24.6%)	26 (30.6%)	51 (28.3%)	
Diagnosis to treatment group (DTI)					
<30 days	14 (53.8%)	31 (44.9%)	31 (36.5%)	76 (42.2%)	
≥30 days	12 (46.2%)	38 (55.1%)	54 (63.5%)	104 (57.8%)	

M = mean, SD = standard deviation, N = number. Statistical tests: ANOVA for continuous variables, Chi-square test for categorical ones.

**Table 4:** The 5-year follow-up.

	No Relapse (N = 155)	Relapse (N = 25)	Total (N = 180)	*p*-Value
**Gender**				
M	146.0 (94.2%)	24.0 (96.0%)	170.0 (94.4%)	0.714 *
F	9.0 (5.8%)	1.0 (4.0%)	10.0 (5.6%)	
**Age**				
Mean (SD)	58.9 (9.2)	59.6 (9.3)	59.0 (9.2)	0.728 **
Range	31.0–76.0	42.0–81.0	31.0–81.0	
**Patient delay**				0.132 *
Unspecified	73.0 (47.1%)	12.0 (48.0%)	85.0 (47.2%)	
˂3 Months	45.0 (29.0%)	11.0 (44.0%)	56.0 (31.1%)	
˃3 Months	37.0 (23.9%)	2.0 (8.0%)	39.0 (21.7%)	
**Histopathologic diagnosis**				0.093 *
SCC	109.0 (70.3%)	13.0 (52.0%)	122.0 (67.8%)	
KSCC	25.0 (16.1%)	4.0 (16.0%)	29.0 (16.1%)	
SCC In Situ	3.0 (1.9%)	2.0 (8.0%)	5.0 (2.8%)	
UCNT	13.0 (8.4%)	3.0 (12.0%)	16.0 (8.9%)	
Others	5.0 (3.2%)	3.0 (12.0%)	8.0 (4.4%)	
**GRADING**				0.055 *
In situ	3.0 (1.9%)	3.0 (12.0%)	6.0 (3.3%)	
G1	24.0 (15.4%)	0.0 (0.0%)	27.0 (15.0%)	
G2	111.0 (71.6%)	14.0 (56.0%)	125.0 (69.4%)	
G3	17.0 (11.0%)	5.0 (20.0%)	22.0 (12.2%)	
**Therapy**				0.027 *
Chemotherapy	18.0 (11.6%)	5.0 (20.0%)	23.0 (12.8%)	
Radiotherapy	110.0 (71.0%)	11.0 (44.0%)	121.0 (67.2%)	
Chemoradiotherapy	27.0 (17.4%)	9.0 (36.0%)	36.0 (20.0%)	
**Emergency**				0.804 *
N-Miss	135.0	21.0	156.0	
Acute Respiratory failure	19.0 (95.0%)	4.0 (100.0%)	23.0 (95.9%)	
Chronic Respiratory failure	1.0 (5.0%)	0.0 (0.0%)	1.0 (4.2%)	
**Stage**				0.321 *
N-Miss	2.0	1.0	3.0	
Stage 1	56.0 (36.6%)	10.0 (41.7%)	66.0 (37.3%)	
Stage 2	28.0 (18.3%)	7.0 (29.2%)	35.0 (19.8%)	
Stage 3	35.0 (22.9%)	2.0 (8.3%)	37.0 (20.9%)	
Stage 4	34.0 (22.2%)	5.0 (20.8%)	39.0 (22.0%)	
**N Stage**				0.722 *
N0	83.0 (53.5%)	14.0 (56.0%)	97.0 (53.9%)	
N1	25.0 (16.1%)	2.0 (8.0%)	27.0 (15.0%)	
N2	13.0 (8.4%)	3.0 (12.0%)	16.0 (8.9%)	
N3	34.0 (21.9%)	6.0 (24.0%)	40.0 (22.2%)	
**T Stage**				0.171 *
T1	52.0 (33.5%)	8.0 (32.0%)	60.0 (33.4%)	
T2	63.0 (40.6%)	10.0 (40.0%)	73.0 (40.6%)	
T3	35.0 (22.6%)	4.0 (16.0%)	39.0 (21.7%)	
T4	4.0 (2.6%)	1.0 (4.0%)	5.0 (2.8%)	
Tis	1.0 (0.6%)	2.0 (8.0%)	3.0 (1.7%)	
**Environment**				
R	71.0 (45.8%)	9.0 (36.0%)	80.0 (44.4%)	0.360 *
U	84.0 (54.2%)	16.0 (64.0%)	100.0 (55.6%)	
**Year**				
2014	29.0 (18.7%)	7.0 (28.0%)	36.0 (20.0%)	0.153 *
2015	24.0 (15.5%)	7.0 (28.0%)	31.0 (17.2%)	
2016	40.0 (25.8%)	7.0 (28.0%)	47.0 (26.1%)	
2017	32.0 (20.6%)	3.0 (12.0%)	35.0 (19.4%)	
2018	30.0 (19.4%)	1.0 (4.0%)	31.0 (17.2%)	
**Tumor site**				
Nasopharynx	23.0 (14.8%)	3.0 (12.0%)	26.0 (14.4%)	0.808 *
Hypopharynx	58.0 (37.4%)	11.0 (44.0%)	69.0 (38.3%)	
Oropharynx	74.0 (47.7%)	11.0 (44.0%)	85.0 (47.2%)	
**Docetaxel**				0.143 *
0	135.0 (87.1%)	19.0 (76.0%)	154.0 (85.6%)	
1	20.0 (12.9%)	6.0 (24.0%)	26.0 (14.4%)	
**Paclitaxel**				0.130 *
0	148.0 (95.5%)	22.0 (88.0%)	170.0 (94.4%)	
1	7.0 (4.5%)	3.0 (12.0%)	10.0 (5.6%)	
**Carboplatin**				0.057 *
0	130.0 (83.9%)	17.0 (68.0%)	147.0 (81.7%)	
1	25.0 (16.1%)	8.0 (32.0%)	33.0 (18.3%)	
**Cisplatin**				0.104 *
0	89.0 (57.4%)	10.0 (40.0%)	99.0 (55.0%)	
1	66.0 (42.6%)	15.0 (60.0%)	81.0 (45.0%)	
**Deceased**				0.509 *
0	132.0 (85.2%)	20.0 (80.0%)	152.0 (84.4%)	
1	23.0 (14.8%)	5.0 (20.0%)	28.0 (15.6%)	

*—Pearson’s Chi-squared test; **—Linear Model ANOVA.

## Data Availability

The original contributions presented in the study are included in the article; further inquiries can be directed to the corresponding author.
